# Supplementary material for: Inhibition of asparagine synthetase effectively retards polycystic kidney disease progression
Source: EMBO Mol Med. 2024 Apr 29;16(6):9. doi: 10.1038/s44321-024-00071-9 (PMC11178866; doi:10.1038/s44321-024-00071-9)
Supplement: Supplementary file 1 — Appendix [file 44321_2024_71_MOESM1_ESM.pdf]

Appendix Information:

Inhibition of Asparagine Synthetase Effectively Retards Polycystic Kidney Disease Progression

Sara Clerici[1]*, Christine Podrini[1,2]*, Davide Stefanoni[1], Gianfranco Distefano[1], Laura Cassina[1], Maria Elena Steidl[1], Laura Tronci[3,4], Tamara Canu[5], Marco Chiaravalli[1], Daniel Spies[1, 6] Thomas A. Bell 3rd[7], Ana S. H. Costa[8,9], Antonio Esposito[5], Angelo D'Alessandro[10], Christian Frezza[11], Angela Bachi[4] and Alessandra Boletta[1]

[1] Molecular Basis of Cystic Kidney Disorders Unit, Division of Genetics and Cell Biology, IRCCS, San Raffaele Scientific Institute, Milan, Italy
[2] Current Address: The BioArte Ltd, Laboratories at Malta Life Science Park (LS2.1.10, LS2.1.12-LS2.1.15), Triq San Giljan, San Gwann, SGN 3000, Malta.
[3] Cogentech SRL Benefit Corporation, 20139 Milan, Italy
[4] IFOM ETS The AIRC Institute of Molecular Oncology; Milan, Italy
[5] Center for Experimental Imaging (CIS), IRCCS, San Raffaele Scientific Institute, Milan, Italy
[6] Center for Omics Sciences (COSR), IRCCS, San Raffaele Scientific Institute, Milan, Italy
[7] Ionis Pharmaceuticals, Carlsbad, California, USA
[8] MRC, Cancer Unit Cambridge, University of Cambridge, Hutchison/MRC Research Centre, Box 197, Cambridge Biomedical Campus, Cambridge, CB2 0XZ, United Kingdom
[9] Current address: Matterworks, Inc. 444 Somerville Avenue, Somerville, 02143 MA, United States.
[10] Department of Biochemistry and Molecular Genetics, University of Colorado Denver, Aurora, CO.
[11] Faculty of Medicine and University Hospital Cologne, Faculty of Mathematics and Natural Sciences, Cluster of Excellence Cellular Stress Responses in Aging-associated Diseases (CECAD), Joseph-Stelzmann-Str. 26-50931 Cologne, Germany
[*] Equal Contributions

Appendix Table of Content:

Appendix Figure S1 2

Appendix Figure S1

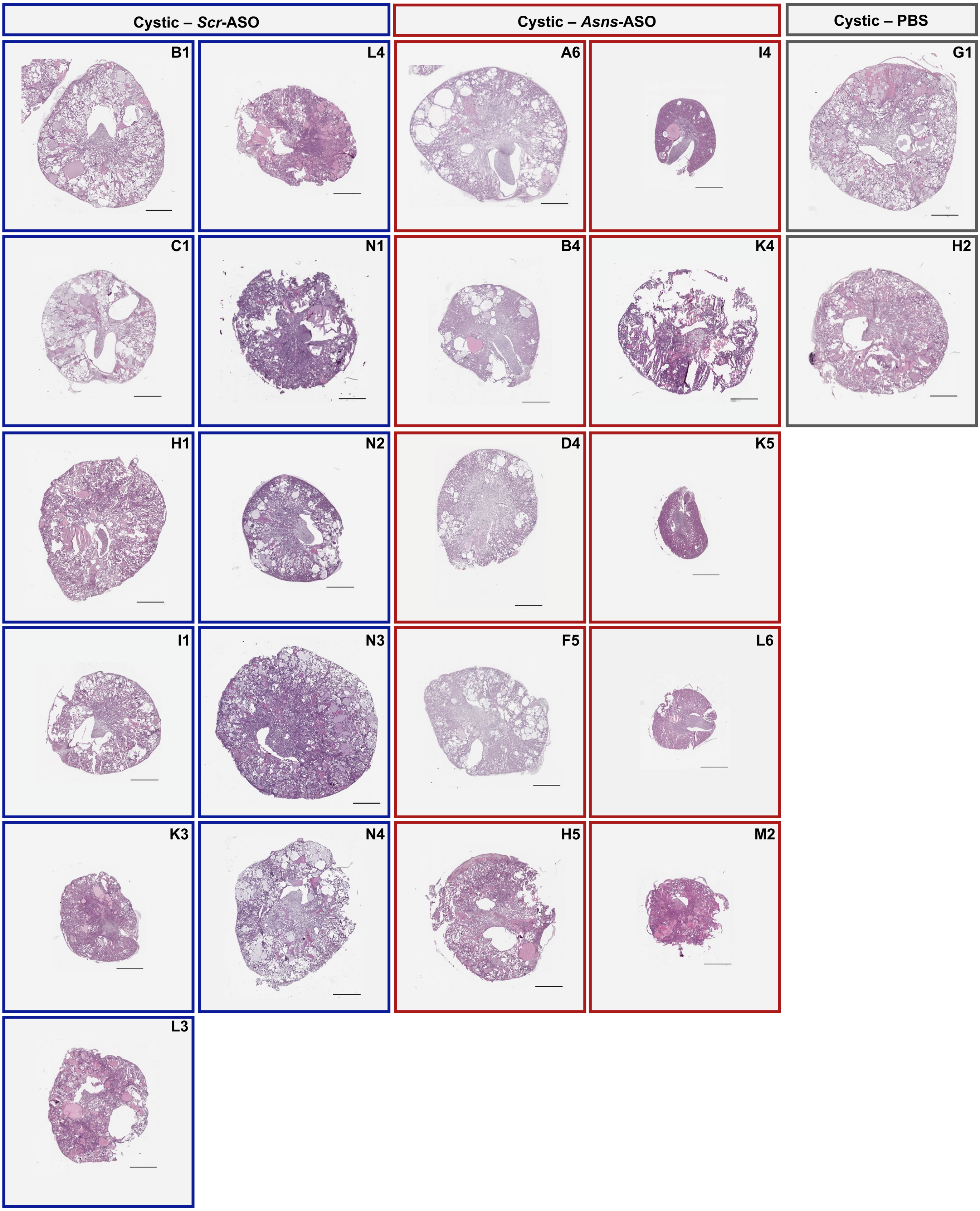

Appendix Figure S1
Hematoxylin and eosin (H&E) of P160 *Tam-Cre;Pkd1*$^{\Delta C/flox}$ cystic kidneys treated with *Scr*-ASO (n=11), *Asns*-ASO (n=10) or PBS (n=2). Scale bar 2 mm. Images B1, I1, L4, B4, I4 and L6 are also presented in Figure 2L.
