# Supplementary figures and images for: Inhibition of asparagine synthetase effectively retards polycystic kidney disease progression
Source: EMBO Mol Med. 2024 Apr 29;16(6):9. doi: 10.1038/s44321-024-00071-9 (PMC11178866; doi:10.1038/s44321-024-00071-9)

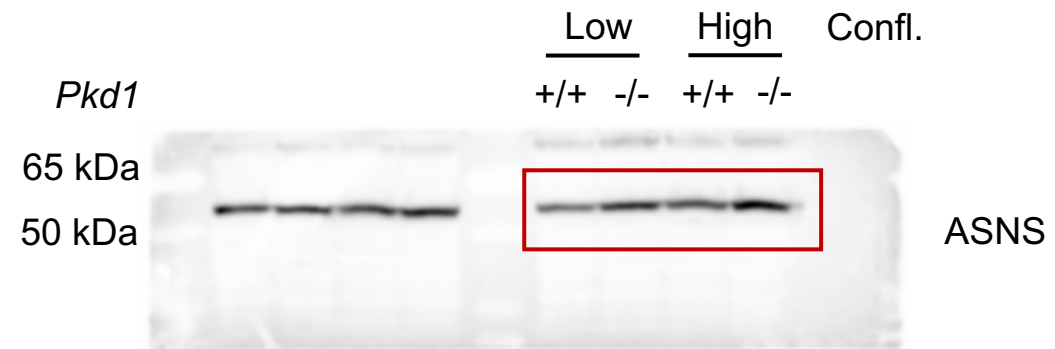

Supplement: Supplementary file 2 — Source data Fig. 1 [file 44321_2024_71_MOESM2_ESM.zip › EMM-2023-18797_SourceDataForFigure1/Figure1C_ASNS.pdf]

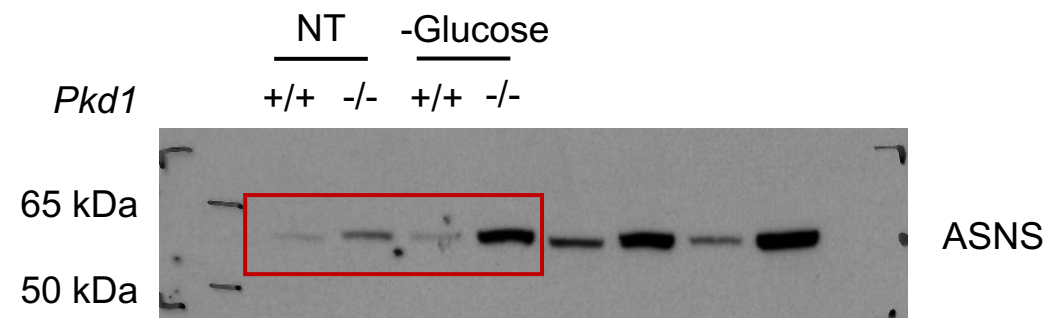

Supplement: Supplementary file 2 — Source data Fig. 1 [file 44321_2024_71_MOESM2_ESM.zip › EMM-2023-18797_SourceDataForFigure1/Figure1B_ASNS.pdf]

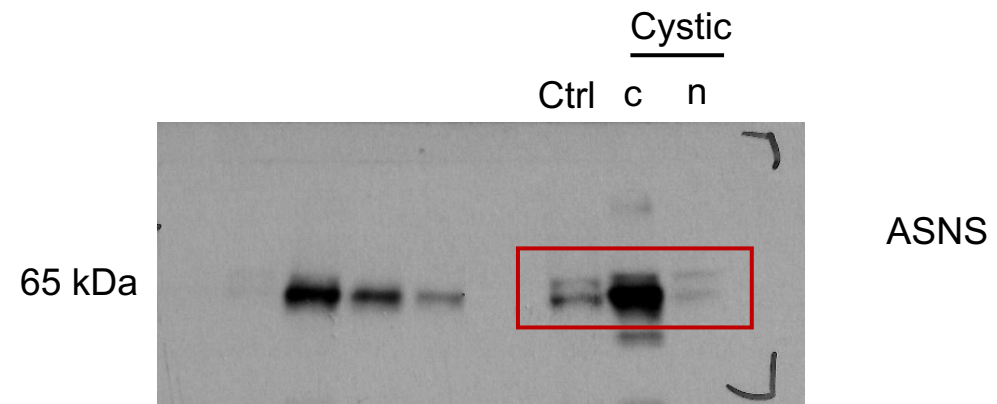

Supplement: Supplementary file 2 — Source data Fig. 1 [file 44321_2024_71_MOESM2_ESM.zip › EMM-2023-18797_SourceDataForFigure1/Figure1D_ASNS.pdf]

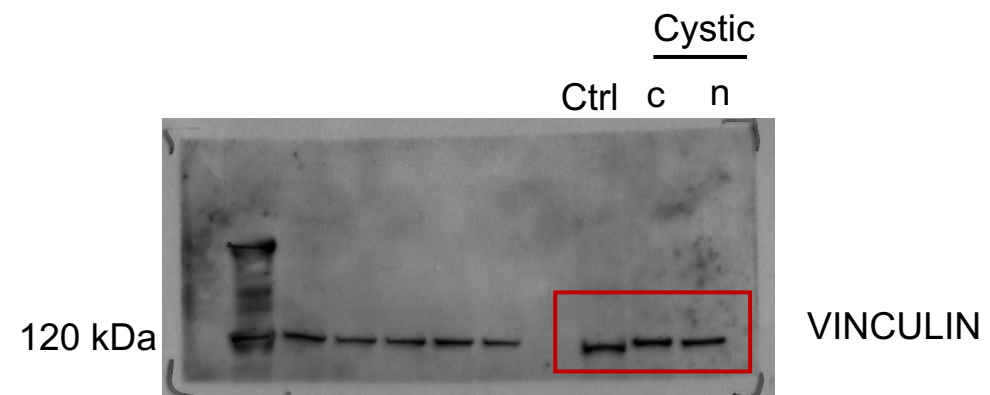

Supplement: Supplementary file 2 — Source data Fig. 1 [file 44321_2024_71_MOESM2_ESM.zip › EMM-2023-18797_SourceDataForFigure1/Figure1D_VINCULIN.pdf]

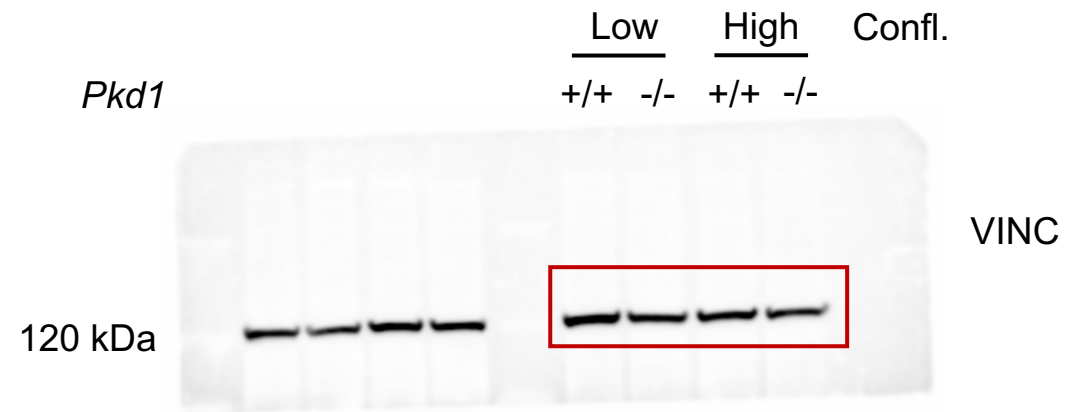

Supplement: Supplementary file 2 — Source data Fig. 1 [file 44321_2024_71_MOESM2_ESM.zip › EMM-2023-18797_SourceDataForFigure1/Figure1C_VINCULIN.pdf]

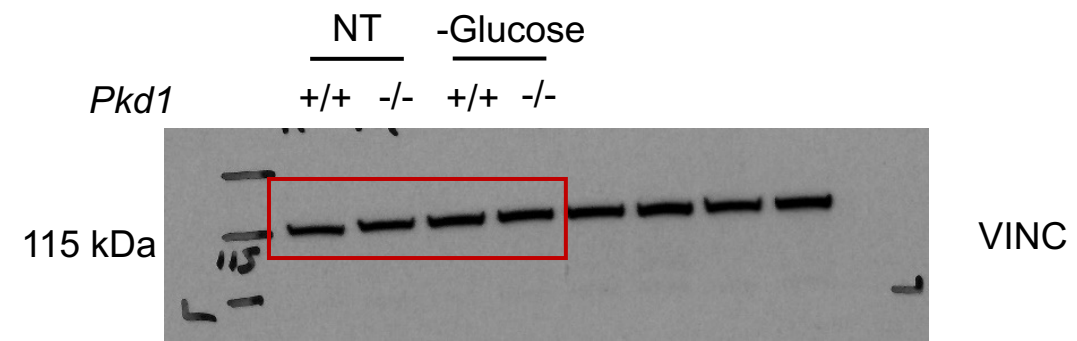

Supplement: Supplementary file 2 — Source data Fig. 1 [file 44321_2024_71_MOESM2_ESM.zip › EMM-2023-18797_SourceDataForFigure1/Figure1B_VINCULIN.pdf]

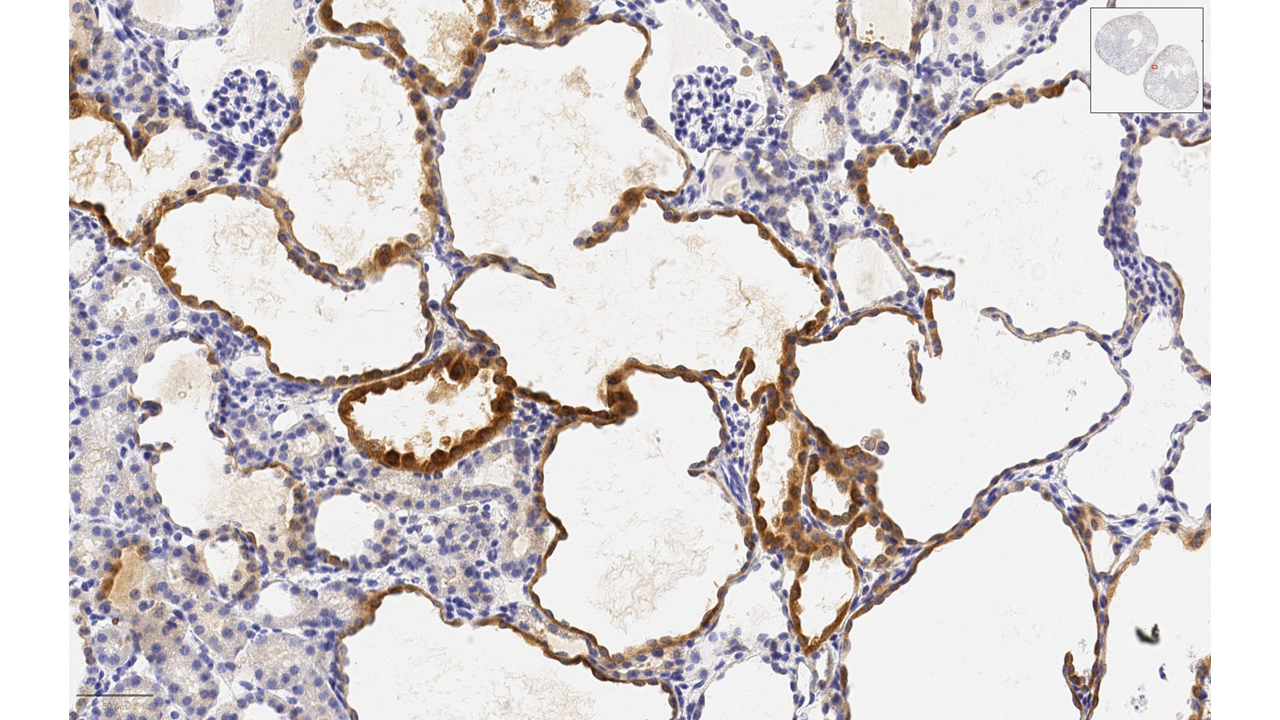

Supplement: Supplementary file 3 — Source data Fig. 2 [file 44321_2024_71_MOESM3_ESM.zip › EMM-2023-18797_SourceDataForFigure2/Figure2D_IHC ASNS_Scr-ASO (P160).tif]

115 kDa

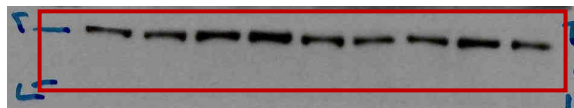

VINC

Supplement: Supplementary file 3 — Source data Fig. 2 [file 44321_2024_71_MOESM3_ESM.zip › EMM-2023-18797_SourceDataForFigure2/Figure2C_VINCULIN.pdf]

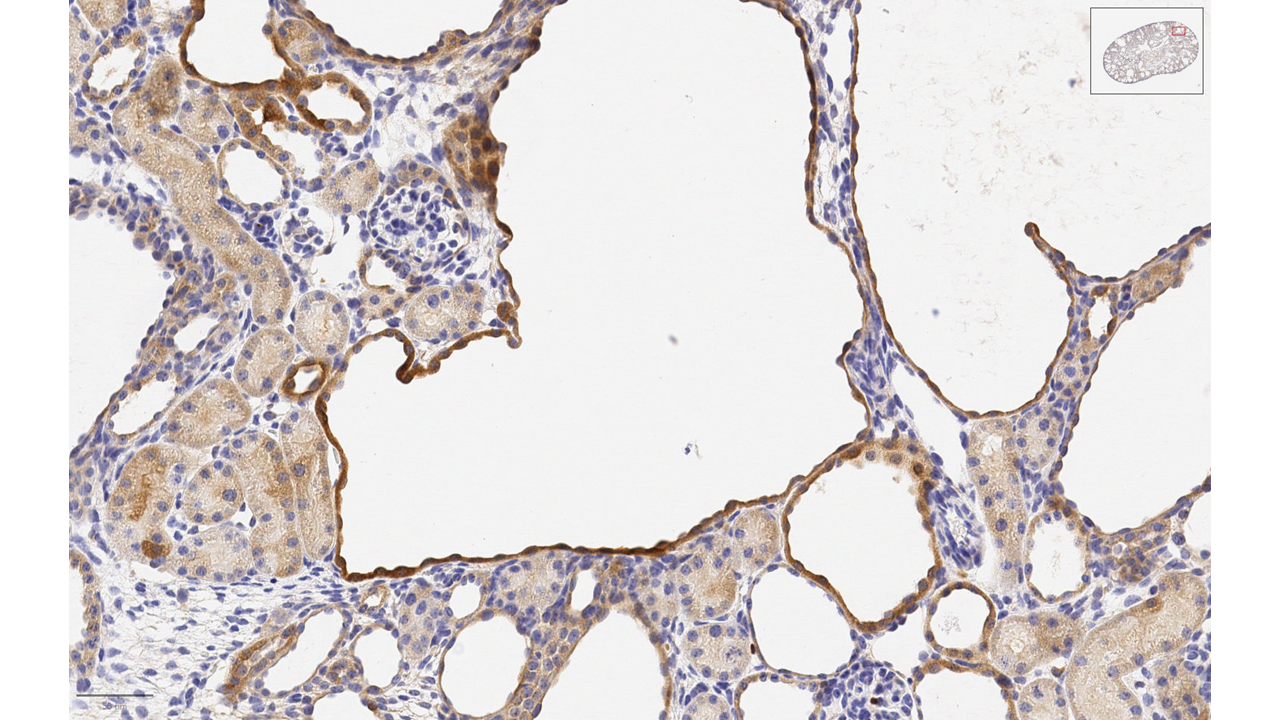

Supplement: Supplementary file 3 — Source data Fig. 2 [file 44321_2024_71_MOESM3_ESM.zip › EMM-2023-18797_SourceDataForFigure2/Figure2D_IHC ASNS_KspCre (P4).tif]

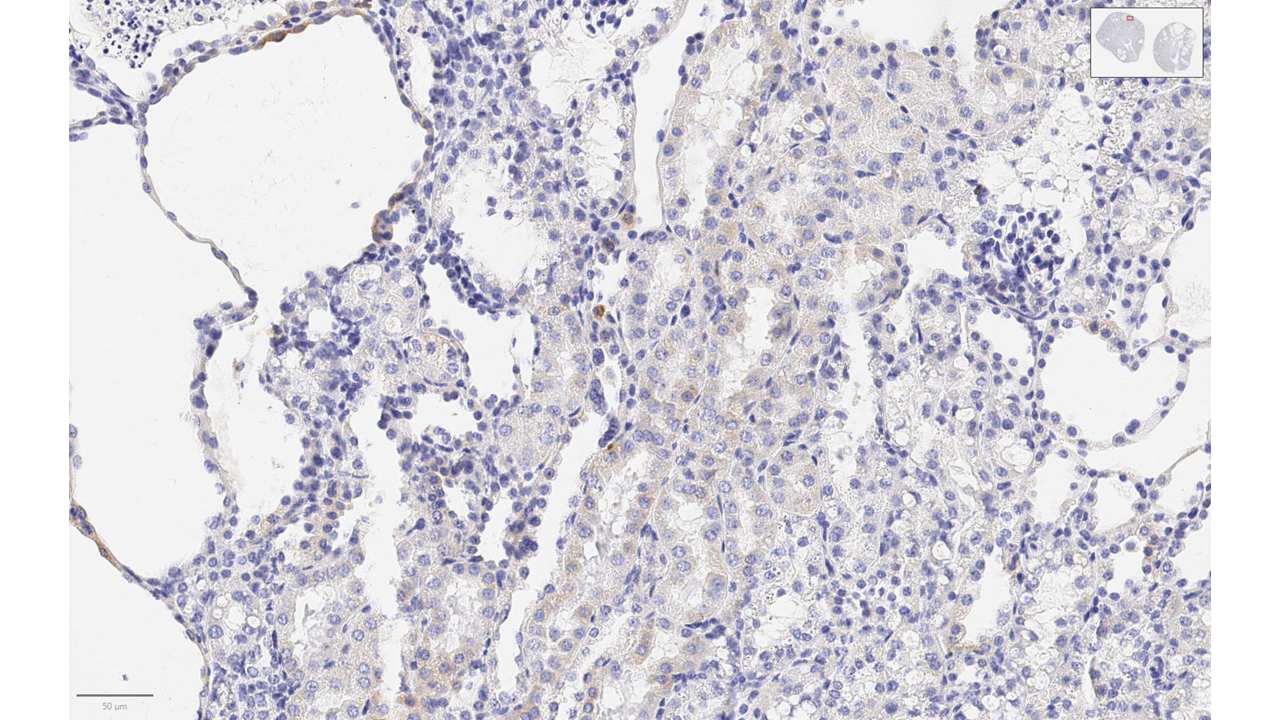

Supplement: Supplementary file 3 — Source data Fig. 2 [file 44321_2024_71_MOESM3_ESM.zip › EMM-2023-18797_SourceDataForFigure2/Figure2D_IHC ASNS_Asns-ASO (P160).tif]

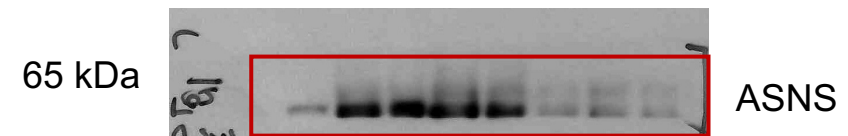

Supplement: Supplementary file 3 — Source data Fig. 2 [file 44321_2024_71_MOESM3_ESM.zip › EMM-2023-18797_SourceDataForFigure2/Figure2C_ASNS.pdf]

65 kDa

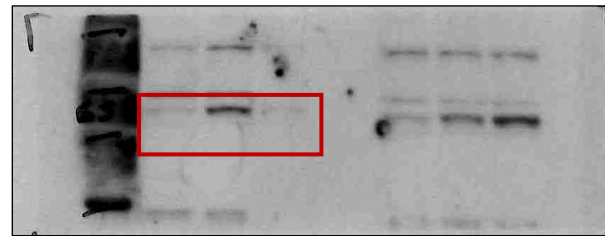

ASNS

Supplement: Supplementary file 4 — Source data Fig. 3 [file 44321_2024_71_MOESM4_ESM.zip › EMM-2023-18797_SourceDataForFigure3/Figure3F_ASNS.pdf]

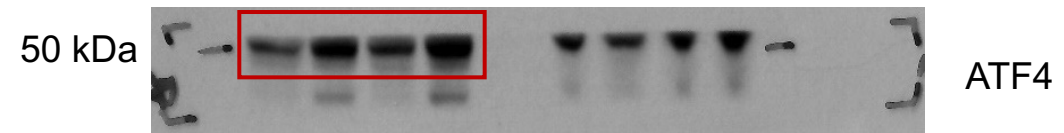

Supplement: Supplementary file 4 — Source data Fig. 3 [file 44321_2024_71_MOESM4_ESM.zip › EMM-2023-18797_SourceDataForFigure3/Figure3B_ATF4.pdf]

VINC

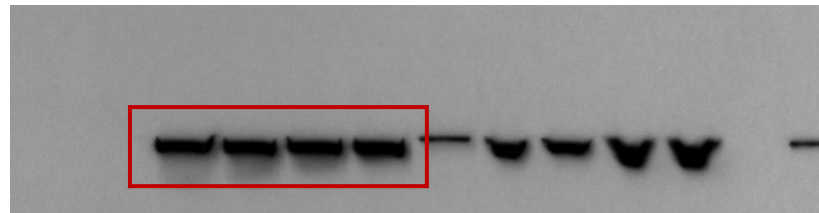

115 kDa

Supplement: Supplementary file 4 — Source data Fig. 3 [file 44321_2024_71_MOESM4_ESM.zip › EMM-2023-18797_SourceDataForFigure3/Figure3B_VINCULIN.pdf]

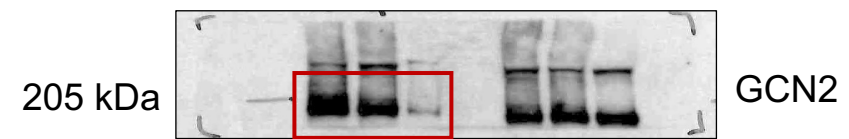

Supplement: Supplementary file 4 — Source data Fig. 3 [file 44321_2024_71_MOESM4_ESM.zip › EMM-2023-18797_SourceDataForFigure3/Figure3F_GCN2.pdf]

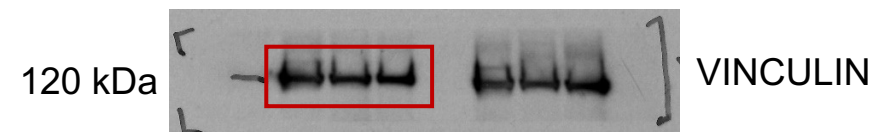

Supplement: Supplementary file 4 — Source data Fig. 3 [file 44321_2024_71_MOESM4_ESM.zip › EMM-2023-18797_SourceDataForFigure3/Figure3F_VINCULIN.pdf]

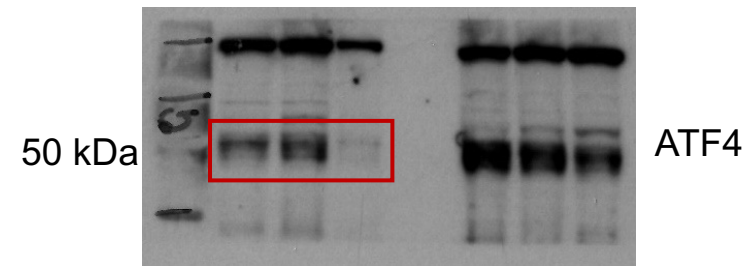

Supplement: Supplementary file 4 — Source data Fig. 3 [file 44321_2024_71_MOESM4_ESM.zip › EMM-2023-18797_SourceDataForFigure3/Figure3F_ATF4.pdf]

205 kDa

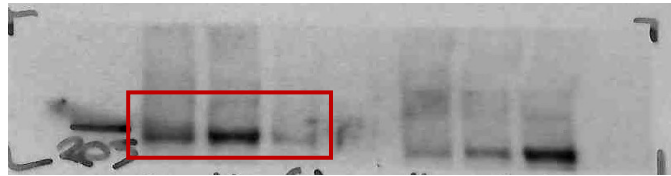

P-GCN2<sup>T899</sup>

Supplement: Supplementary file 4 — Source data Fig. 3 [file 44321_2024_71_MOESM4_ESM.zip › EMM-2023-18797_SourceDataForFigure3/Figure3F_P-GCN2.pdf]

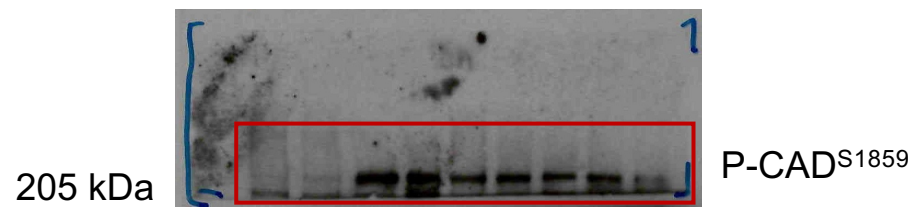

Supplement: Supplementary file 7 — Source data Fig. 6 [file 44321_2024_71_MOESM7_ESM.zip › EMM-2023-18797_SourceDataForFigure6/Figure6A_P-CAD.pdf]

115 kDa

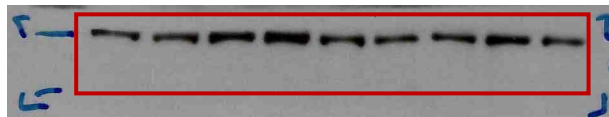

VINC

Supplement: Supplementary file 7 — Source data Fig. 6 [file 44321_2024_71_MOESM7_ESM.zip › EMM-2023-18797_SourceDataForFigure6/Figure6A_VINCULIN.pdf]
