# Supplementary material for: Inhibition of asparagine synthetase effectively retards polycystic kidney disease progression
Source: EMBO Mol Med. 2024 Apr 29;16(6):9. doi: 10.1038/s44321-024-00071-9 (PMC11178866; doi:10.1038/s44321-024-00071-9)
Supplement: Supplementary file 10 — Expanded View Figures [file 44321_2024_71_MOESM10_ESM.pdf]

Expanded View Figures

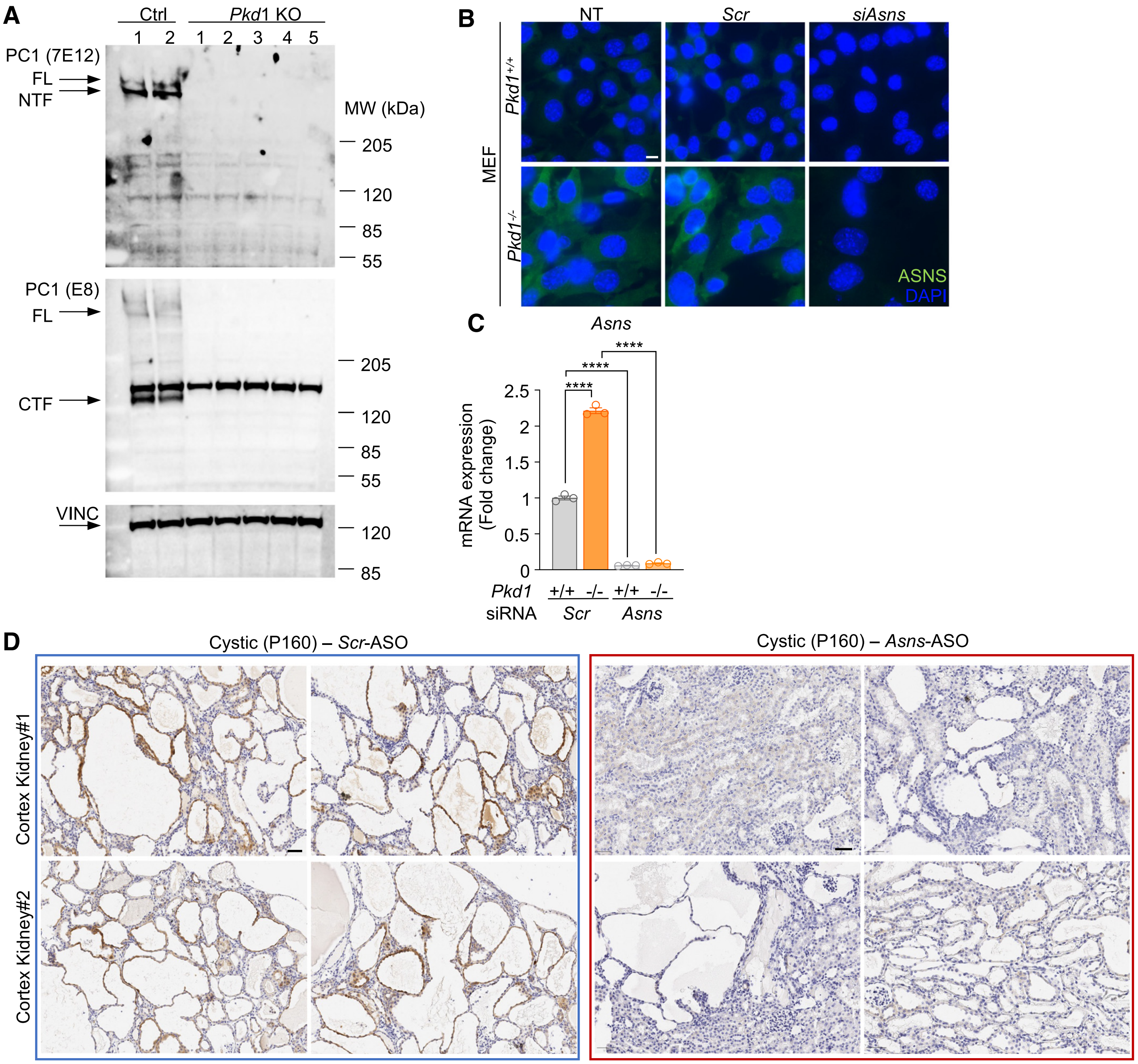

Figure EV1. Validation of cell lines KO for Pkd1 and of antibodies directed against ASNS.

(**A**) Immunoblotting on control and *Pkd1* KO mCCD clones for the detection of PC1 protein, using PC1 7E12 and E8 antibodies. FL, PC1 full-length protein; NTF, PC1 N-terminal fragment; CTF, PC1 C-terminal fragment. PC1 bands were detected in control cells and not in *Pkd1* KO clones. Vinculin was used as loading control to show equal loading of protein samples. (**B**) Representative images of immunofluorescence staining of ASNS in $Pkd1^{-/-}$ and $Pkd1^{+/+}$ MEF cells, untreated (NT), scrambled (*Scr*) or silenced for *Asns*. Scale bar (10 μm). (**C**) *Asns* expression in $Pkd1^{-/-}$ and control MEF cells upon silencing ($n = 3$ biological replicates). (**D**) Representative images of IHC ASNS staining in cystic renal epithelium of *Tam-Cre;*$Pkd1^{\Delta C/flox}$ (P160) mice treated with *Scr*-ASO or *Asns*-ASO. Scale bar (50 μm). Data information: In (**C**) data are shown as mean ± SD. One-way ANOVA, corrected with Tukey's multiple comparisons. ****$P < 0.0001$.

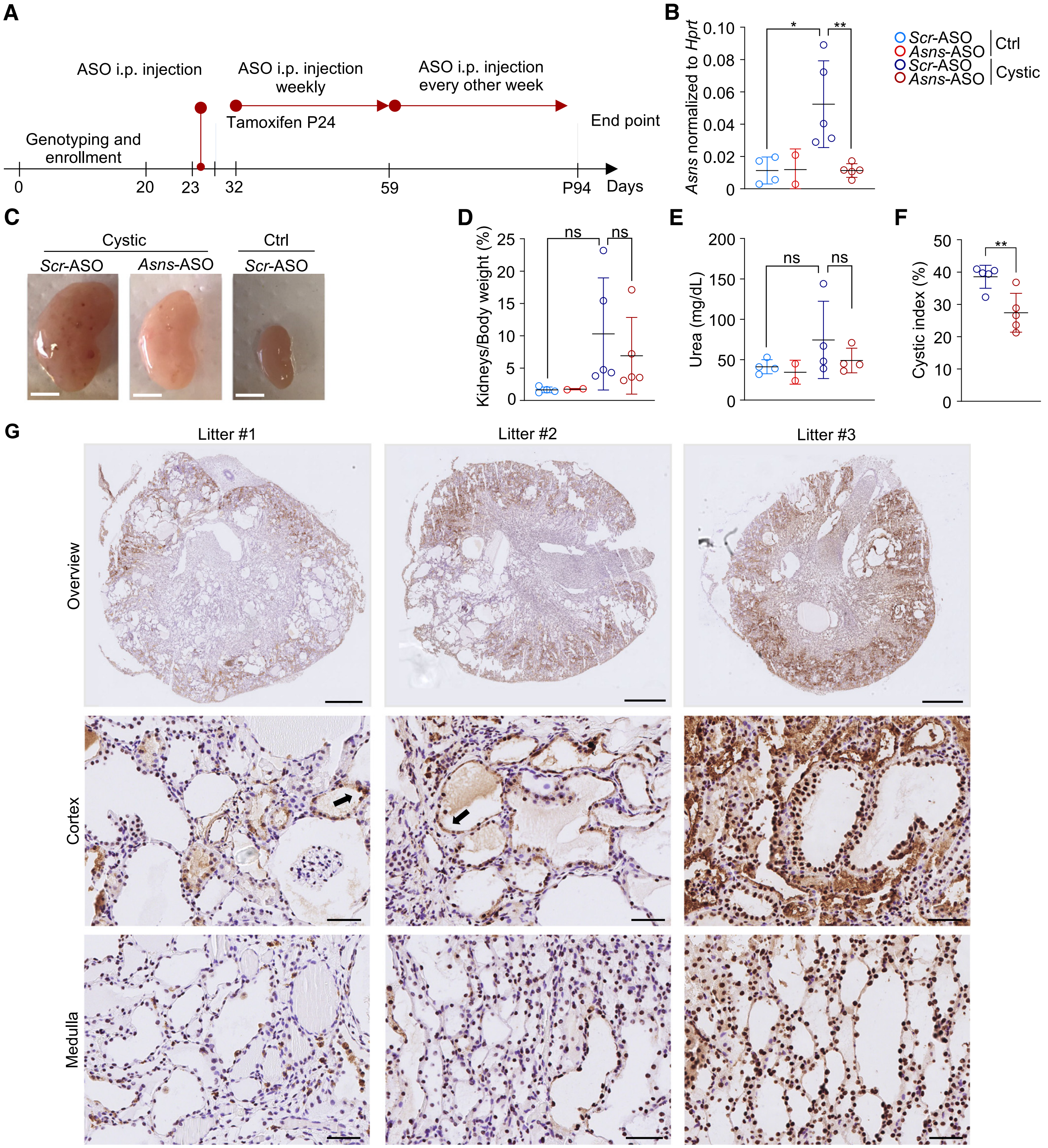
A
ASO i.p. injection
ASO i.p. injection weekly
ASO i.p. injection every other week
Tamoxifen P24
End point
Genotyping and enrollment
0
20
23
32
59
P94
Days
B
Asns normalized to Hprt
Scr-ASO
Asns-ASO
Ctrl
Scr-ASO
Asns-ASO
Cystic
C
Cystic
Ctrl
Scr-ASO
Asns-ASO
Scr-ASO
D
Kidneys/Body weight (%)
ns
ns
E
Urea (mg/dL)
ns
ns
F
Cystic index (%)
G
Litter #1
Litter #2
Litter #3
Overview
Cortex
Medulla

© The Author(s)

◀ **Figure EV2. Pilot test of Asns-ASO on a medium-term PKD model.**

(**A**) Experimental design of pilot study on *Tam-Cre;Pkd1*$^{\Delta C/flox}$ and relative controls treated with *Asns*-ASO or *Scr*-ASO ($n = 4$ ctrl *Scr*-ASO; $n = 2$ ctrl *Asns*-ASO; $n = 5$ cystic *Scr*-ASO; $n = 5$ cystic *Asns*-ASO). (**B**) *Asns* mRNA expression in *Tam-Cre;Pkd1*$^{\Delta C/flox}$ and relative controls treated with *Asns*-ASO or *Scr*-ASO ($n = 4$ ctrl *Scr*-ASO; $n = 2$ ctrl *Asns*-ASO; $n = 5$ cystic *Scr*-ASO; $n = 5$ cystic *Asns*-ASO). (**C**) Representative images of cystic kidneys and relative controls at P94 treated with *Asns*-ASO or *Scr*-ASO. (**D**) Percentage of kidneys weight normalized to body weight of cystic and relative controls treated with *Asns*-ASO or *Scr*-ASO ($n = 4$ ctrl *Scr*-ASO; $n = 2$ ctrl *Asns*-ASO; $n = 5$ cystic *Scr*-ASO; $n = 5$ cystic *Asns*-ASO). (**E**) BUN of cystic and relative control kidneys treated with *Asns*-ASO or *Scr*-ASO ($n = 4$ ctrl *Scr*-ASO; $n = 2$ ctrl *Asns*-ASO; $n = 4$ cystic *Scr*-ASO; $n = 4$ cystic *Asns*-ASO). (**F**) Quantification of the cystic area percentage of the total kidney area measured in transversial sections of cystic *Scr*-ASO or *Asns*-ASO groups ($n = 5$ cystic *Scr*-ASO; $n = 5$ cystic *Asns*-ASO). (**G**) ASO distribution in cystic kidneys harvested from *Asns*-ASO-treated mice at P95 from three different litters. Representative images of ASO distribution in total kidneys (upper panel, scale bar (1 mm)), renal cortex middle panel, scale bar (50 µm)), and renal medulla (lower panel, scale bar (50 µm)). Arrows indicate the ASO-positive cystic epithelium. Data information: in (**B**, **D**, **E**) data are shown as mean ± SD. One-way ANOVA. ns non-significant; $^{*}P < 0.05$; $^{**}P < 0.01$. In (**F**) data are shown as mean ± SD. Student's unpaired two-tailed *t* test. $^{**}P < 0.01$.

© The Author(s)

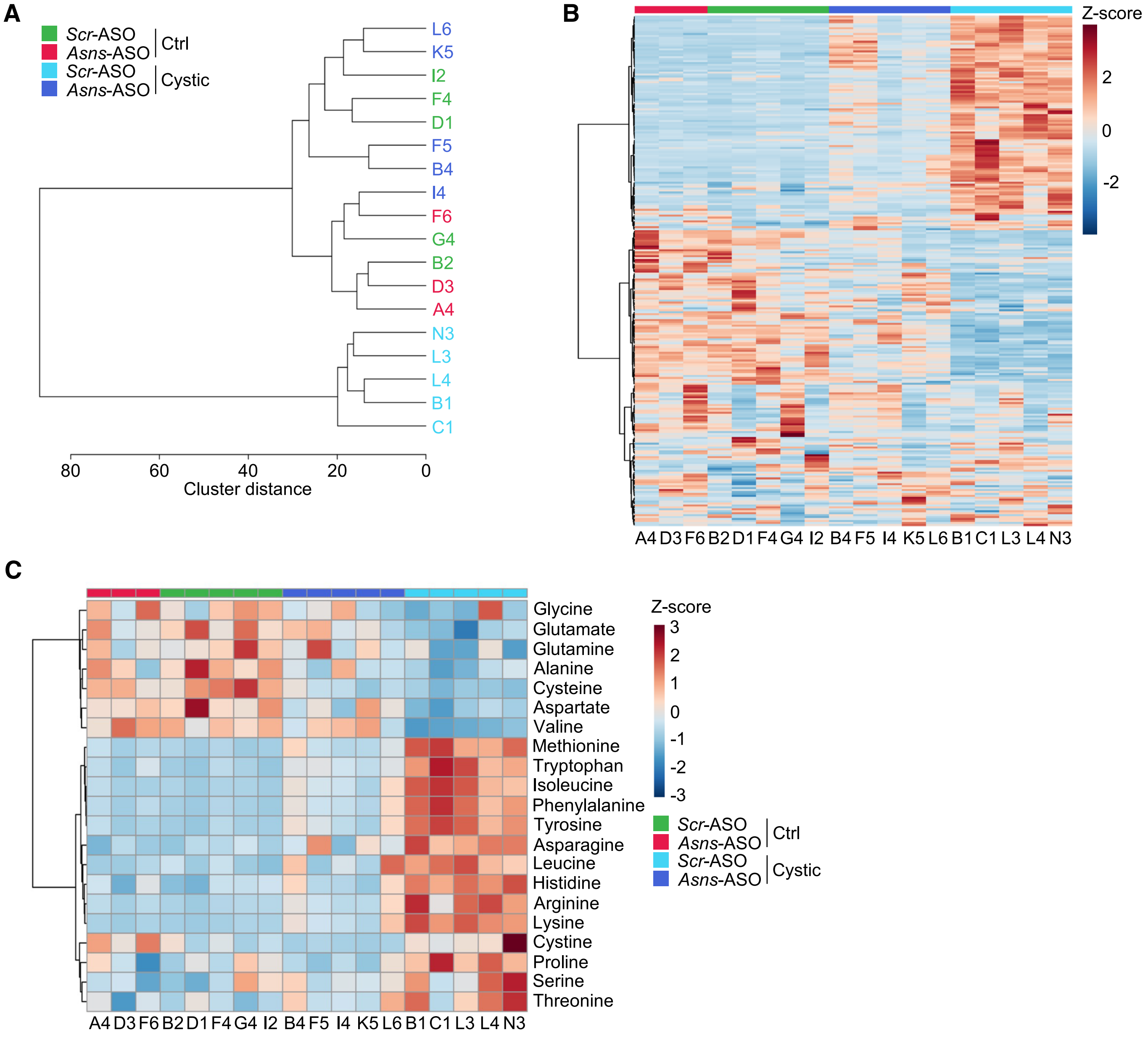

Figure EV3. Hierarchical clustering of metabolomics data.

(**A**) Dedrogram diagram showing the hierachical clustering of 4 ASO-treated groups of samples analyzed through LC–MS. (**B**) Heatmap based on the HCA of the metabolom (265 metabolites) comparing *Scr*- and *Asns*-ASO-treated cystic and control kidneys. (**C**) Heatmap based on the HCA of amino acids detected in PKD cystic and control kidneys treated with *Scr*-ASO or *Asns*-ASO. Data information: in (**A–C**) clustering based on t test/ANOVA result performed with Metaboanalyst 5.0.

© The Author(s)

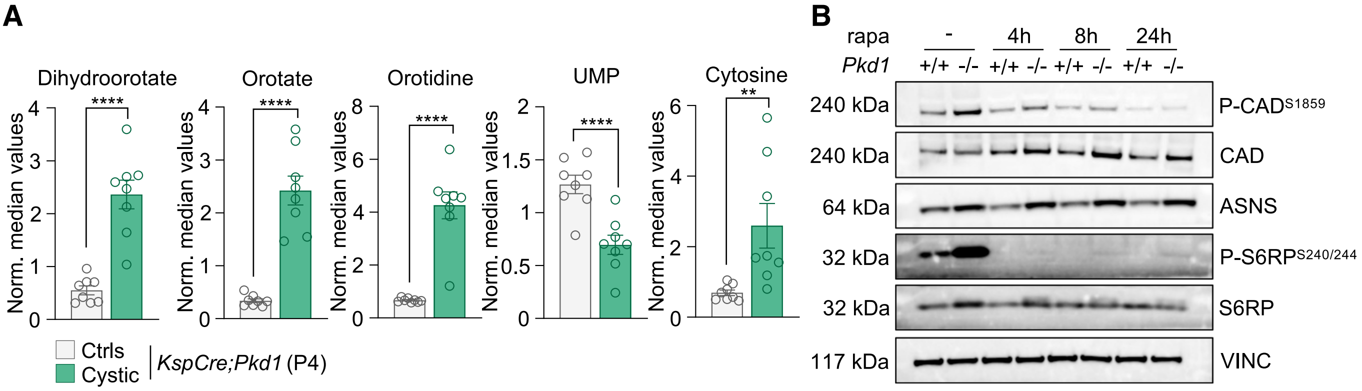

Figure EV4. Validation of the CAD-pyrimidine biosynthesis pathway in different models.

(**A**) Intermediate metabolites of de novo pyrimidine biosynthesis pathway analyzed through untargeted metabolomics of *KspCre;Pkd1*$^{\Delta C/flox}$ cystic kidneys and relative controls at P4 ($n = 8$ ctrls; $n = 8$ cystic). (**B**) P-CAD, ASNS and P-S6RP protein expression in *Pkd1*$^{-/-}$ and control MEF cells treated for 4 h, 8 h, or 24 h with rapamycin (50 nM), after overnight serum starvation ($n = 3$). Data information: in (**A**) data are shown as mean ± SEM. Student's *t* test. $^{**}P < 0.01$; $^{****}P < 0.0001$.

© The Author(s)
