# Supplementary material for: Inhibition of asparagine synthetase effectively retards polycystic kidney disease progression
Source: EMBO Mol Med. 2024 Apr 29;16(6):9. doi: 10.1038/s44321-024-00071-9 (PMC11178866; doi:10.1038/s44321-024-00071-9)
Supplement: Supplementary file 4 — Source data Fig. 3 [file Figure3C_ATF4_VINCULIN.pdf › EMM-2023-18797_SourceDataForFigure3/Figure3C_ATF4_VINCULIN.pdf]

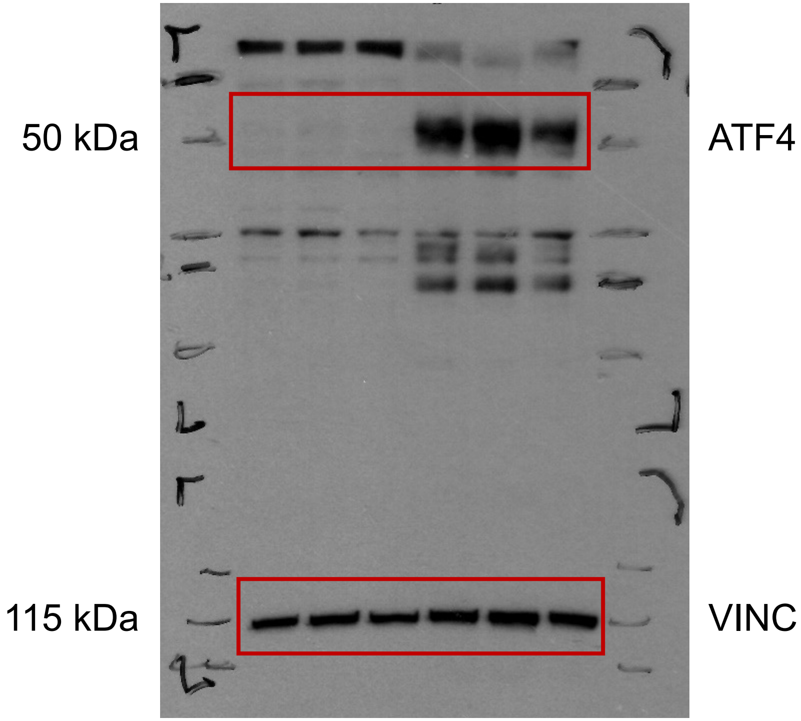
50 kDa
ATF4
115 kDa
VINC
